# Post-Traumatic-Related Technical Errors in Orthopantomographic Imaging

**DOI:** 10.3390/medicines9120063

**Published:** 2022-12-09

**Authors:** Oana Almășan, Raluca Ancuța Roman, Mihaela Hedeşiu, Simion Bran, Sara Roman, Bianca Petric, Cristian Dinu

**Affiliations:** 1 Department of Prosthetic Dentistry and Dental Materials, Iuliu Hațieganu University of Medicine and Pharmacy, 32 Clinicilor Street, 400006 Cluj-Napoca, Romania; 2 Department of Maxillofacial Surgery and Radiology, Oral Radiology, Iuliu Hațieganu University of Medicine and Pharmacy, 32 Clinicilor Street, 400006 Cluj-Napoca, Romania; 3 Department of Maxillofacial Surgery and Implantology Oral and Maxillofacial Surgery, Iuliu Hațieganu University of Medicine and Pharmacy, 37 Iuliu Hossu Street, 400029 Cluj-Napoca, Romania

**Keywords:** panoramic radiography, error source, jaw fracture

## Abstract

**Background:** This study aimed at identifying errors encountered in orthopantomography (OPG) in post-traumatic patients caused by limitations in performing a correct technique. **Methods:** A retrospective observational study was performed. Diagnosis, exposure/processing mistakes, positioning-related errors, and bimaxillary immobilization were evaluated. **Results:** Thirty panoramic radiographs with mandible fractures were examined. Twelve error types were encountered: errors in exposure or processing, air radiolucency in the palatoglossal space, errors in the alignment of the Frankfort horizontal plane: head in flexion, with a joyful expression or head extended, with a somber appearance, errors towards the mid-sagittal plane (lateral head inclination, deviation, or rotation), errors caused by the non-use of the bite-block or inappropriate position on the device, errors caused by positioning outside the focal plane, artifacts/shadow images produced by post-operative metal plates, and bimaxillary immobilization errors. The number of errors per radiograph ranged from two to a maximum of five. The most dominant ones were inappropriate alignment in the focal plane and lateral rotation of the head in over 70% of cases. Lateral deviation and palatoglossal air were present in more than 50% of images. **Conclusions:** In trauma cases, technical difficulties in obtaining a proper OPG image are common and often insurmountable, limiting the diagnosis.

## 1. Introduction

In panoramic radiography, also known as orthopantomography (OPG), positioning issues are relatively frequent and lead to the acquisition of images of poor quality [1]. An unsatisfactory OPG may result in a false diagnosis and, thus, an ineffective treatment strategy [2]. To assist patients to avoid one of the most frequent patient positioning errors, a radiolucent band of air in the palatoglossal space due to not sustaining the tongue to the roof of the mouth, a breathing procedure has been introduced that can enhance the diagnostic utility of OPG [3]. An error in head placement during panoramic imaging could cause the patient’s improper location in the Frankfort horizontal plane [4].

Panoramic radiography is the most conducted extra-oral imaging examination in dental radiology [5]. It offers information on the teeth as well as the periodontium, sinuses, temporomandibular joint, or soft tissues. It enables the evaluation of the structures of interest with minimum radiation exposure and optimum operating time [6]. The OPG and cone-beam computed tomography (CBCT) are common imaging modalities, mostly for trauma indications [7]. Immediate post-reduction imaging is a common procedure in treating mandibular fractures [8]. However, CBCT is more trustworthy than OPG and avoids planning inaccuracy [9]. For mandible fractures, suspicions of computed tomography (CT), and OPG scans are frequently indicated, with CT having minimal extra benefit compared to OPG in emergency circumstances, such as mandible fractures or oral injuries [10]. The radiation doses produced by dental imaging procedures can be found on a web dose calculator [5].

As an operator-dependent technology, radiology and imaging specialists must first have adequate training and then identify and eliminate the factors that cause errors in the final image. They are primarily responsible for maintaining a high degree of quality so that the diagnosis and treatment are carried out accurately.

The most common flaws that occur on OPG that the operator may prevent are connected to improper positioning or subsequent movement when the patient does not fully follow the professional’s instructions. Removing metallic items is the operator’s responsibility: earrings, necklaces, and clips generate artifacts on radiographs, obscuring anatomical elements or even pathologies, and ultimately altering image quality. To minimize overexposure or underexposure, the exposure parameters, represented by milliamperes and kilovoltage, must be adjusted for each patient based on age, weight, and head size. If the digital images are slightly overexposed, post-processing enables the reduction of the contrast and hence the adjustment of the density, which is also true in the opposite circumstance.

This study aimed at identifying errors encountered in panoramic radiographs of trauma patients, establishing their sources, classifying them, and triggering a warning to draw attention to the need the technician and radiologist are to expect and, if possible, prevent these errors.

## 2. Materials and Methods

A retrospective, analytical, and observational study was performed. The research was conducted by analyzing the panoramic radiographs performed in a maxillofacial surgery service in patients with trauma by the same experienced radiographer during a half-year period between January 2020 and June 2020 using Villa Strato X 200 Digital panoramic equipment. A high-quality viewing system was used. The Iuliu Hațieganu University of Medicine and Pharmacy ethics committee approved the research (approval number 125.05.17).

### Selection and Description of Participants

Thirty panoramic radiographs with fractures were evaluated. The inclusion criteria were the presence of traumatic mandible fractures on panoramic radiography before or after treatment. The exclusion criteria were all other diagnoses for which orthopantomograms were conducted. The errors assessed were exposure/processing errors, the use or absence of the bite-block, air band in the palatoglossal space, positioning, and alignment with the laser-beam-related errors. Errors related to bimaxillary immobilization were also noted. The positioning errors related to the mid-sagittal plane were classified as lateral deviation, lateral rotation, and lateral-inclination errors, those related to the Frankfort plane alignment were head in flexion and head in extension, respectively, and those classified as “positioning outside the focal plane” were those related to canine laser beam alignment.

The patient data and outcomes were processed and statistically analyzed using SPSS software. The Wilson score method was used to calculate confidence intervals for proportions from the total number of cases and the Fischer exact test. Error frequency on images was determined as the number of images presenting a certain error. The relative error frequency represented the percentage from the total number of errors encountered, and a 95% confidence interval was calculated for the frequency of the error on the number of images studied. 

## 3. Results

**Figure 1:**
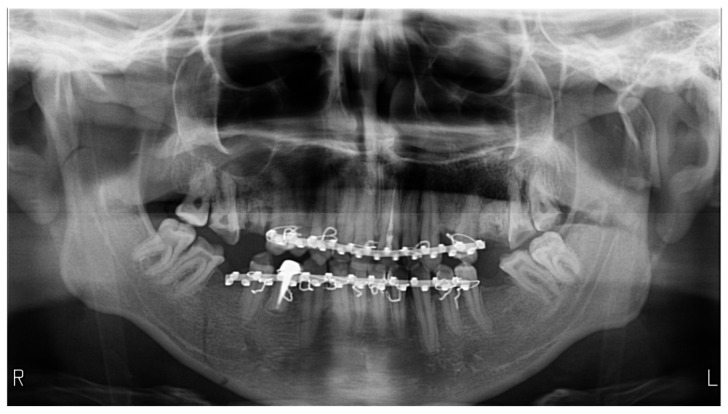
Panoramic radiography exemplifies the air present in the palatoglossal space as a radiolucent band. The patient presents a triple fracture of the mandible, bilateral low subcondylar and the body on the right side, with bimaxillary immobilization.

**Figure 2:**
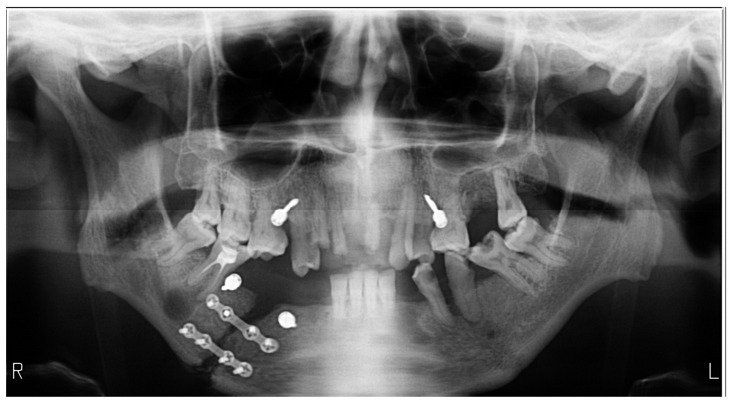
Orthopantomography of a case with a double fracture of the mandible, left subcondylar, and lateral on the right side, the later surgically reduced and fixed, exemplifies the head in flexion error, with poor visibility in the menton region.

**Figure 3:**
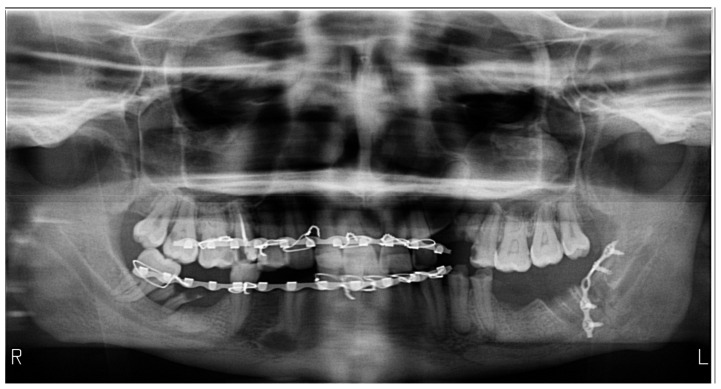
Orthopantomography with a sad appearance, a blurred frontal region, produced by the head extension position, in a patient treated surgically for a fracture of the left angle of the mandible. The asymmetry between the right and left sides with a larger hemi-mandible on the right side is produced by a lateral deviated position of the head towards the left side. Additionally, superior apical portions in the frontal region are obscured by an air band in the palatoglossal space. The bite-block was not used due to bimaxillary immobilization.

**Figure 4:**
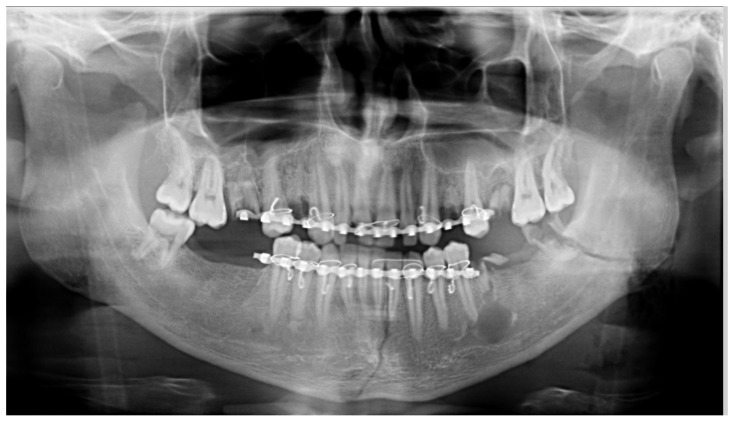
Orthopantomography in a patient with a double mandible fracture presents a positioning error, with lateral rotation of the head towards the left side and, consequently, an obvious asymmetry in the image. The errors are caused by the impossibility of using the bite-block when applying bimaxillary immobilization.

**Figure 5:**
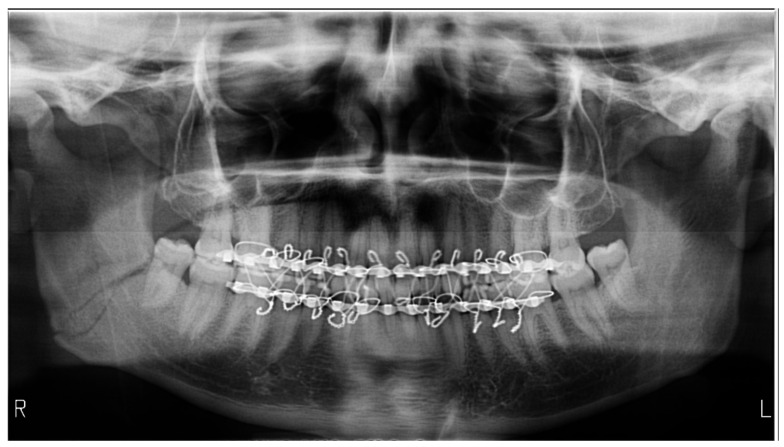
Orthopantomography of a patient with a fracture on the right angle of the mandible; bimaxillary immobilized exemplifies an improper adjustment in the focal through due to the absence of the bite-block, resulting in a blurry contour of the teeth, more obvious in the frontal region. Additionally, the image shows a head position in extension and incorrect exposure factors.

Each radiograph contains one or more errors. The number of errors per radiograph ranged from two to five (Table 1). Following errors were encountered: errors in exposure or processing, the radiolucent band of air in the palatoglossal space produced by the tongue’s non-adhesion to the hard palate (Figure 1), errors aligning the Frankfort horizontal plane: head in flexion, with a joyful expression or head extended (Figure 2), with an unfavorable appearance (Figure 3), errors in aligning the mid-sagittal plane (lateral head inclination, lateral head deviation or lateral head rotation) (Figure 4), errors caused by the absence of the bite-block (Figure 3, Figure 4 and Figure 5) or inappropriate bite on the device, errors caused by positioning the jaw outside the focal plane (Figure 3 and Figure 5), artifacts/opaque images produced by post-operative metallic plates, bimaxillary immobilization-related errors (Figure 5 and Table 2).

The bite-block was used in eight images that presented lateral head deviation or rotation, showing no statistical significance for the lateral head rotation. The images without the presence of the bite-block showed a lateral deviation of 86.36%, with a *p*-value of 0.003 in the Fischer exact test (Table 3).

## 4. Discussion

The analysis of panoramic radiographs was chosen because it is the most common diagnostic imaging test requested by dentists and is also an examination prone to errors if sufficient attention is not paid to the positioning. The presence of trauma due to tumefaction of the soft parts makes it difficult to correctly align the head’s standard planes with the equipment’s laser beams.

Multiple errors were detected, with the most frequent being related to improper alignment of the canine line, producing a lateral rotation of the head, usually towards the affected part. This also appears if the use of the bite-block is impossible. Commonly, patients with fractures are first immobilized in the emergency room if the fracture is mobile and then sent to the radiographer to confirm and to completely evaluate the case. Bimaxillary-immobilized patients cannot open their mouths, so the impossibility of using a bite-block is present. This makes it difficult to align the arches in the frontal plane. Due to either an anterior or a posterior location of the chin, a distorted and imprecise aspect of the teeth appears. Metallic artifacts were the least encountered errors. The high percentage of the palatoglossal air presence is explained by the impossibility of holding the tongue on the palate due to pain, as a fracture of the mandible is accompanied by pain at any traction of the oral floor muscles.

It has been shown that OPG has inadequacies that can affect its use, including low resolution, uneven magnification, and geometric deformation [11]. The most frequent placement errors in OPG were reported as being the positioning of the patient’s head, tongue, or chin [2]. Therefore, the patient must be positioned correctly to obtain panoramic images with high image resolution [12].

A study by Kaviani et al. showed that errors in patient orientation were the most common type of errors detected on panoramic radiographs [13], which is consistent with our study’s findings. In our study, the frequency of the lateral-rotation error was only observed at 36.6%, comparable to their result of 39.5% [13]. Depending on the number of fracture lines, the tumefaction of the face may sometimes be unilateral or bilateral. When unilateral, these situations are more prone to appear and are hardly managed by the radiographer.

In the study by Dhillon M. and the authors, the radiolucent band at the apex of the maxillary teeth was the most frequent error, occurring in 55.7% of the cases [14]; however, in our research, it appeared in 66.66% of instances. The head extension was present in 17.9% and flexion in 16.2% of cases, which are considerably lower than in our study, which recorded 26.66% and 36.66%, respectively. Their study’s frequency of out-of-focal plane radiographs was 48.3%, while we obtained a percentage of 70% [14]. Our explanation would be the absence of a bite-block and a chin limit present in other equipment to limit a more anterior position. Another situation for out-of-focal through position is when tumefaction is very ample, the patient is immobilized, and the arch position and shape under the soft parts are hard to appreciate by the radiographer. This shows the importance of taking sufficient time when performing OPG in these typical situations and a good experience in working with trauma cases.

In a different investigation, 75 OPG errors were assessed in edentulous patients; the results revealed that head extension error (41.3%) was higher than in our study by more than 14%, but out-of-focal plane positioning (34.7%) was significantly lower than our values (70%). It is to specify that the mentioned study did not study trauma patients [15].

A relatively high rate of errors was encountered in our study, which is consistent with other research. Therefore, due to the relatively high error rates in panoramic radiography, more training for the professionals doing these investigations is needed [16]. According to the research, the tongue repositioning operation can significantly reduce tongue position errors during panoramic radiography, with substantial modifications in tongue shadow being observed [17]. Gross et al. reported that the majority of the panoramic images which they investigated had some technical issues, and image augmentation could prevent the need for retakes [18]. To decrease the frequency of common operator errors seen on OPG, there is a need to implement dental radiography training for all operators [19]. When a possible inaccuracy is suspected, alternatives for adapting the approach should be identified.

The present study considered only patients with diagnosed fractures and trauma-induced clinical modification as potential sources of errors in technique, comparing it with previous studies assessing errors in panoramic imaging in general.

Since their clinical presentation makes it challenging for the radiographer to estimate the skeletal morphology in patients with mandible fractures, orthopantomography is recommended in these cases. This estimation is essential to correctly align the arches with the equipment’s focal plane. The number of flaws in the image increases as a result of the pain level that was experienced and the challenges that came with adhering to the technical instructions. Operator training requires knowledge of error-generating situations when performing OPG in trauma cases. In sum, their corrective alternatives need to be known, as well as the existence of situations in which the patient’s condition does not allow for the performance of a perfect technique, as there are sources of errors independent of the radiographer’s skills.

### 4.1. Strengths and Limitations

This study showed that many errors occur when performing panoramic imaging in trauma cases, which are related to the impossibility of correct positioning of the head. These errors combine since some derive from another; they must be expected and ideally precluded. The small sample size and brief observation period may represent some of this study’s shortcomings. However, the significant number of inaccuracies (twelve) seen in this small batch of panoramic radiography may help radiographers maintain control of how maxillofacial trauma is addressed and how its image limits interfere with the diagnosis, thereby improving the patient’s condition.

### 4.2. Implications for Practice and Future Research

The awareness of errors in OPG should be accounted for to avoid retaking radiography. Attention to patient positioning, allocating sufficient time for the procedure, and correctly adjusting the exposure factors to a tumefied area to enhance clarity, could be considered a solution to avoid low image quality. Types of equipment with special chin blocks designed for trauma/immobilized arches would prevent at least the out-of-focal situation. Future prospective studies are encouraged to gather data on the inaccuracies in panoramic radiography for maxillofacial trauma over longer observation periods with more cases.

## 5. Conclusions

Each radiography in the examined group presented errors, ranging from two to a maximum of five per radiography. The commonest ones were due to a decreased appreciation of the arch position under tumefied soft parts. Combinations of errors are a fact in these situations since one misalignment generates an incorrect alignment with the next laser beam. To minimize unnecessary patient exposure and radiography repetition in front of a trauma case, it is essential to be aware of the potential for errors to occur during panoramic imaging acquisition.

A careful approach and radiographer awareness can increase the accuracy and quality of panoramic radiography, along with a decrease in radiation exposure.

## Figures and Tables

**Table 1:** The number of errors on a single image.

Number of Errors on a Single OPG	Total Errors (*n*)	Relative Frequency (%)	Relative Frequency (CI95%)
2	3	6.6	[01.85–21.32]
3	12	40	[24.59–57.68]
4	10	33.33	[19.23–5122]
5	4	13.33	[05.31–29.68]
6	1	03.33	[00.59–16.67]

CI95%—confidence interval 95%.

**Table 2:** Encountered errors in panoramic radiography.

Diagnostic	Total Errors (*n*)	Error Frequency on Images (%)	Relative Error Frequency (%)	Frequency on Images (CI95%)
Exposure errors	24	80	53.33	[62.69–90.49]
Palatoglossal air	20	66.66	52.63	[48.78–80.77]
Head in flexion	11	36.66	39.28	[21.87–54.49]
Head in extension	8	26.66	44.44	[14.18–44.45]
Lateral head inclination	18	60	45	[42.32–75.41]
Lateral head deviation	18	60	52.94	[42.32–75.41]
Lateral head rotation	11	36.66	73.33	[21.87–54.49]
Absence of bite-block	28	93.33	51.85	[78.68–98.15]
Inappropriate bite	2	6.66	66.66	[01.85–21.32]
Positioning outside the focal plane	21	70	46.66	[52.12–83.34]
Radiographs with bimaxillary immobilization errors	23	76.66	62.16	[59.07–88.21] *

CI95%—confidence interval 95%, * *p* < 0.01 Fischer test.

**Table 3:** Lateral head positioning errors concerning the use of the bite-block.

Presence of Bite-Block	Total Errors (*n*) %	Relative Frequency (CI95%)	Absence of Bite-Block (*n*) %	Relative Frequency (CI95%)
Lateral rotation	(3) 37.50	[13.68–69.43]	(10) 45.45	[26.92–65.34]
Lateral deviation	(2) 25.00	[07.15–59.07]	(19) 86.36	[66.67–95.25] *
Total	8		22	

* *p* < 0.01 Fischer test.

## Data Availability

The data included in this research are available upon reasonable request from the corresponding author.
